# Abnormal CSF amyloid-β42 and tau levels in hip fracture patients without dementia

**DOI:** 10.1371/journal.pone.0204695

**Published:** 2018-09-25

**Authors:** Esther S. Oh, Kaj Blennow, George E. Bigelow, Sharon K. Inouye, Edward R. Marcantonio, Karin J. Neufeld, Paul B. Rosenberg, Juan C. Troncoso, Nae-Yuh Wang, Henrik Zetterberg, Frederick E. Sieber, Constantine G. Lyketsos

**Affiliations:** 1 Departments of Medicine, Johns Hopkins University School of Medicine, Baltimore, MD, United States of America; 2 Department of Psychiatry and Behavioral Sciences, Johns Hopkins University School of Medicine, Baltimore, MD, United States of America; 3 Department of Pathology, Johns Hopkins University School of Medicine, Baltimore, MD, United States of America; 4 Clinical Neurochemistry Lab, Institute of Neuroscience and Physiology, Department of Psychiatry and Neurochemistry, the Sahlgrenska Academy at the University of Gothenburg, Mölndal, Sweden; 5 Harvard Medical School, Boston, MA, United States of America; 6 Department of Medicine, Beth Israel Deaconess Medical Center, Boston, MA, United States of America; 7 Aging Brain Center, Hebrew SeniorLife, Boston, MA, United States of America; 8 Department of Biostatistics, Johns Hopkins University School of Medicine, Baltimore, MD, United States of America; 9 Department of Epidemiology, Johns Hopkins University School of Medicine, Baltimore, MD, United States of America; 10 Department of Molecular Neuroscience, UCL Institute of Neurology, Queen Square, London, United Kingdom; 11 UK Dementia Research Institute at UCL, London, United Kingdom; 12 Department of Anesthesiology and Critical Care Medicine, Johns Hopkins University School of Medicine, Baltimore, MD, United States of America; Texas Technical University Health Sciences Center, UNITED STATES

## Abstract

**Background:**

There is strong association of Alzheimer’s disease (AD) pathology with gait disorder and falls in older adults without dementia. The goal of the study was to examine the prevalence and severity of AD pathology in older adults without dementia who fall and sustain hip fracture.

**Methods:**

Cerebrospinal fluid (CSF) was obtained from 168 hip fracture patients. CSF Aβ42/40 ratio, p-tau, and t-tau measures were dichotomized into normal vs. abnormal, and categorized according to the A/T/N classification.

**Results:**

Among the hip fracture patients, 88.6% of the cognitively normal (Clinical Dementia Rating-CDR 0; n = 70) and 98.8% with mild cognitive impairment (CDR 0.5; n = 81) fell in the abnormal biomarker categories by the A/T/N classification.

**Conclusions:**

A large proportion of older hip fracture patients have CSF evidence of AD pathology. Preoperative determination of AD biomarkers may play a crucial role in identifying persons without dementia who have underlying AD pathology in perioperative settings.

## Introduction

Alzheimer’s disease (AD) is the most common cause of dementia in the United States (U.S.), with an estimated 5.5 million affected individuals in 2017. The annual incidence of AD is expected to double by 2050, because of the increased number of older adults [1]. A further indicator of the significant public health impact of AD and related dementias is the annual economic cost estimated at upwards of 215 billion dollars in the U.S. [2].

Physical disability, with associated falls, is a significant contributor to AD related health care costs [1]. One of the consequences of falls is hip fracture, with up to 97% of hip fractures occurring as the result of a fall [3]. As with AD the number of hip fractures in adults 65 years and older is increasing in the U.S., and is expected to approach 300,000/year by 2030 in the U.S [4]. Hip fracture is associated with a multitude of complications including prolonged rehabilitation, loss of independence, and one-year mortality of 26% [5]. The economic cost associated with hip fracture is also high, with annual Medicare expenditures of 2.9 billion dollars [6].

One of the major risk factors for both falls and hip fractures is gait disorder [3]. In the past several years, much new evidence has accumulated elucidating the association between AD pathology and gait disorders in persons without dementia. In one study, the presence of AD pathology at autopsy was associated with more rapid rate of decline in walking speed several years prior to death, independent of dementia [7]. More recently, amyloid-beta (Aβ) burden measured by positron emission tomography (PET) has linked Aβ pathology with worse performance on multiple gait parameters, in cross-sectional and longitudinal studies of older adults with normal cognition or mild cognitive impairment (MCI) [8–10]. A prospective study of cognitively normal older adults has also reported that imaging and CSF biomarkers suggestive of underlying AD pathology were associated with faster time to first fall [11].

The exact mechanisms by which AD pathology is associated with gait disorders remain unclear. However, one study demonstrated an association between gait disorder and Aβ burden in striatum, especially the posterior putamen which receives its primary input from motor and sensorimotor cortices [8]. More recently, a study examining sensorimotor integration in AD patients with motor disturbance suggested a link with Aβ pathology and the cholinergic system, which is a major contributor of motor function [12].

Despite the mounting evidence above, few studies have examined the prevalence of AD pathology in hip fracture patients who arguably suffer one of the most serious complications of gait disorders. The goal of this study was to examine a cohort of hip fracture patients for underlying AD pathology as evidenced by CSF biomarkers, and to determine how often such pathology is seen in hip fracture patients without dementia.

## Materials and methods

### Participants

The study comprised 168 consecutive hip fracture patients enrolled in the randomized clinical trial “A Strategy to Reduce the Incidence of Postoperative Delirium in Elderly Patients” (STRIDE) who had preoperative Clinical Dementia Rating (CDR) assessments completed [13]. Detailed study description has been published [13,14]. Briefly, inclusion criteria were age ≥65, preoperative Mini-Mental State Exam (MMSE) score ≥15, and eligible for spinal anesthesia. Main exclusion criteria were preoperative delirium, stage IV congestive heart failure, or severe chronic obstructive pulmonary disease. Informed consent was obtained from patients, or their appropriate legal representatives for patients unable to give informed consent due to cognitive impairment. The Johns Hopkins Medical Institution’s Institutional Review Board oversaw the trial.

### Study procedures

Demographic data were collected from patients, informants, and medical records. Prior to surgery, trained research staff obtained history from the patients and their informants. The research staff also administered the MMSE to the patients and the Short Form of the Informant Questionnaire on Cognitive Decline in the Elderly (Short IQCODE) to the family or caregivers [15]. A consensus panel of two psychiatrists and one geriatrician blinded to the intervention scored the CDR, which is a modification of the previously published CDR [16]. The CDR scoring was based on assessment of all available clinical and cognitive data, as well as the Short IQCODE [15] and other history collected from the patient and the informant prior to surgery.

CSF samples were collected at the onset of the routine spinal anesthesia, aliquoted and stored at –80 ^o^C. Previously unthawed CSF samples were analyzed for Aβ40 and Aβ42, phosphorylated tau (p-tau), and total tau (t-tau) at the Clinical Neurochemistry Laboratory of the Sahlgrenska University Hospital, Mölndal, Sweden. Aβ40 and Aβ42 was assayed using MSD electrochemiluminescence assay (Meso Scale Discovery, Rockville, MD, USA), and p-tau and t-tau were assayed using INNOTEST enzyme-linked immunosorbent assays (Fujirebio, Ghent, Belgium) according to the manufacturer’s specifications. Assays were run with standardized internal controls to account for inter-assay variability by board-certified laboratory technicians who were blinded to clinical data. All performed within plate approval limits from the lab quality manger (QM) program including intra-assay coefficient of variation (CV) below 10%. Apolipoprotein E (APOE) was genotyped at the Johns Hopkins Alzheimer’s Disease Research Center (JHADRC).

### Statistics

Classification cutoff using mixture modeling was used to determine the abnormal biomarker level of Aβ pathology as previously had been done [17,18]. The optimal cutoff ratio of CSF Aβ42/Aβ40, which correlates well with abnormal amyloid PET [19], was determined to be ≤ 0.8 (CSFAβ42/40 ratio x 10) based on a population based study conducted in the same laboratory. The biomarker of tau pathology was categorized as normal if CSF p-tau was < 60 pg/ml or abnormal if ≥ 60 pg/ml, and biomarker of neuronal degeneration or neuronal injury was categorized as normal if CSF t-tau was ≤ 350 pg/ml or abnormal if > 350 pg/ml. These cutoff values were based on previous studies that utilized similar platforms and procedures as the current study [20] and also validated in a hip fracture population [21]. Distribution of abnormal CSF biomarkers were described based on CDR scores.

In individuals without dementia (CDR 0 or 0.5), CSF biomarkers were further divided into categories according to the A/T/N classification system, where “A” refers to the value of an Aβ biomarker, “T” the value of a tau biomarker, and “N,” the value of a neurodegeneration biomarker [22]. In this study, CSF Aβ42/40 is classified as normal (A-) or abnormal (A+), p-tau as normal (T-) or abnormal (T+), and t-tau as normal (N-) or abnormal (N+) based on the cutoff values above. The A/T/N classifications were also mapped to the corresponding existing National Institute on Aging-Alzheimer’s Association (NIA-AA) criteria as it had been previously done [23]. Distributions of these categories were described according to participants’ age categories.

Baseline demographics were compared among different CDR groups using Χ^2^ or Fisher’s exact test for dichotomous variables and one-way ANOVA F-test or Kruskal-Wallis (non-parametric) test for continuous variables. The proportion of abnormal biomarker levels were compared among different CDR groups using Χ^2^ test for dichotomous variables. P values ≤ 0.05 were regarded as statistically significant. The required sample size was calculated based on the hypothesized intervention effect (light vs. heavy anesthesia sedation) of the parent study “A Strategy to Reduce the Incidence of Postoperative Delirium in Elderly Patients” (STRIDE) [13]. Therefore, we evaluated the minimal detectable effect size for the between group differences of mean biomarker levels across the CDR groups based on the predetermined CDR group sizes from the STRIDE trial. The minimal detectable effect size for the between group differences of mean biomarker levels across the CDR groups was 0.24 using an ANOVA F-test with CDR subgroup sizes of 70, 81, and 17, respectively, alpha level of 0.05, and power of 0.80. Analyses were conducted using STATA 14.2 (StataCorp, College Station, TX) and GraphPad Prism 7.00 (La Jolla, California USA).

## Results

Mean patient age was 81.9 (SD 7.8), with the largest group in the ≥ 85 year old group. About three quarters of the patients were white females. The majority of patients were either cognitively normal (CDR 0) or had mild cognitive impairment (MCI) (CDR 0.5) (Table 1). There was a significant difference in CSF t-tau levels between the three CDR groups (p = 0.01), but not in CSF Aβ42 (p = 0.39) or p-tau (p = 0.10) levels. All biomarker ratios including Aβ42/40, Aβ42/p-tau, and Aβ42/t-tau differed significantly across CDR groups. *APOE* genotyping showed that 24% of the patients had at least one copy of *APOE*-ε4. The largest proportion of *APOE*-ε4 carriers was in the CDR ≥ 1 group (Table 2).

**Table 1:** Baseline clinical data by Clinical Dementia Rating (CDR) categories.

	Clinical Dementia Rating (CDR)				
	Total	0	0.5	≥1^a^	P-value
	(n = 168)	(n = 70)	(n = 81)	(n = 17)	
Demographics					
Age, yrs, mean (SD)	81.9(7.75)	78.5(7.02)	84.1(6.99)	85.8(8.85)	**<0.001**
Sex, male, n (%)	44(26)	15(21)	21(26)	8(47)	0.10
female, n (%)	124(74)	55(79)	60(74)	9(53)	
Race, n (%)					
Nonwhite race or Hispanic	6(3.6)	2 (2.9)	3(3.7)	1(5.9)	0.70
Education^b^, n (%)	44(26)	24(34)	18(22)	2(12)	0.09
MMSE, mean (SD)	24.3(3.75)	26.6(2.55)	23.5(3.06)	19.0(4.02)	**<0.001**

Abbreviations: Years (yrs); Mini-Mental State Examination (MMSE).

^a^ CDR ≥ 1 included CDR 1 (n = 13) and CDR 2 (n = 4).

^b^ ≥ college—attended some years of college or above.

**Table 2:** Baseline CSF data by Clinical Dementia Rating (CDR) categories.

	Clinical Dementia Rating (CDR)				
	Total	0	0.5	≥ 1	p-value
	(n = 168)	(n = 70)	(n = 81)	(n = 17)	
**Laboratory Values**					
**CSF**, mean (SD)					
Aβ 42 (pg/ml)	297.16(161.20)	316.57(151.07)	285.99(165.19)	270.46(182.5 7)	0.39
Aβ 40 (pg.ml)	5032.94(1799.75)	5049.14(1802.80)	5021.73(1765.64)	5019.68(2050.03)	0.99
Aβ 42/40^a^	0.59(0.20)	0.63(0.19)	0.56(0.19)	0.52(0.22)	**0.03**
p-tau (pg/ml)^b^	56.62(25.35)	51.75(20.93)	59.42(28.52)	63.00(23.51)	0.10
t-tau (pg/ml)	493.21(282.33)	419.78(195.70)	535.48(336.88)	594.20(236.42)	**0.01**
Aβ42/t-tau	0.71(0.38)	0.84(0.37)	0.65(0.35)	0.50(0.36)	**<0.001**
Aβ42/p-tau	5.75(2.81)	6.59(2.72)	5.28(2.65)	4.53(3.11)	**0.003**
	Total				
**APOE**^c^	(n = 158)	(n = 67)	(n = 76)	(n = 15)	p-value
APOE-ε4^d^, n (%)	38(24.1)	16(23.9)	16(21.1)	6(40.0)	0.29

Cerebrospinal fluid (CSF); Amyloid-beta (Aβ); total tau (t-tau); phosphorylated tau (p-tau).

^a^The Aβ42/Aβ40 ratio was calculated as Aβ42/Aβ40 x 10 as previously has been done [20].

^b^One subject in the CDR 0 group had p-tau below the detection limit.

^**c**^APOE genotyping was available in 158 subjects.

^d^APOE-ε4 denotes those who had at least one copy of APOE-ε4 (APOE-ε4+/ APOE-ε4+) or (APOE-ε4+/ APOE-ε4-). Percentage (%) is calculated as number of individuals with at least one copy of APOE-ε4/subgroup total. Ninety six percent (23/24) of the patients with at least one copy of APOE-ε4 had abnormal Aβ biomarker levels by Aβ 42/40 ratios.

**Fig 1:**
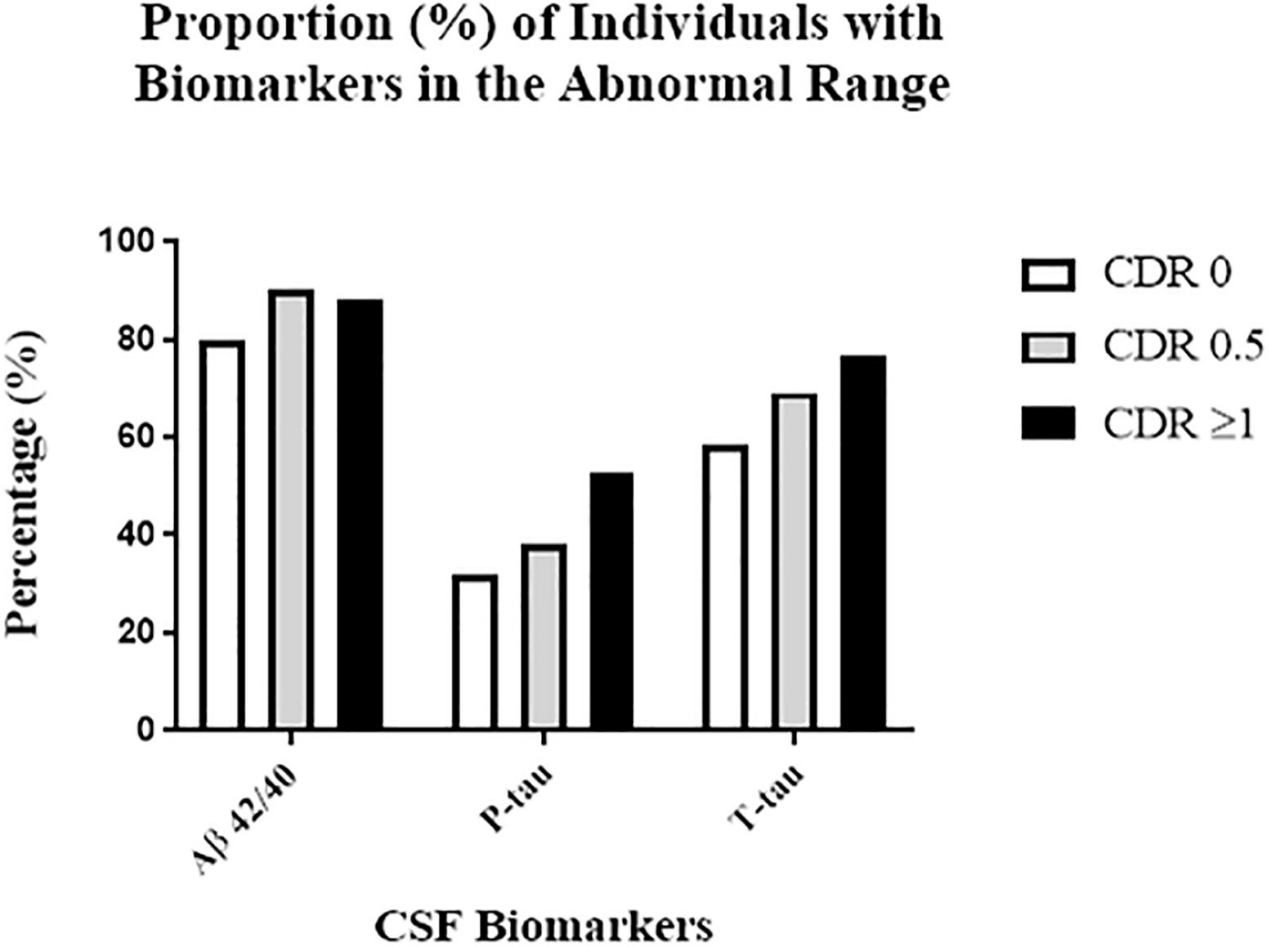
High proportion of individuals with CSF biomarkers in the abnormal range in hip fracture population. Proportion of individuals with biomarkers in the abnormal range for the entire cohort (total) and by CDR categories. CDR 0 (n = 70), CDR 0.5 (n = 81), CDR ≥ 1 (n = 17) CSF Aβ42/40 ratio (cutoff ≤ 0.8): Total– 85.7% (144/168); CDR 0–80% (56/70); CDR 0.5–90.1% (73/81); CDR ≥ 1–88.2% (15/17), (Χ^2^ = 3.24, df = 2, p = 0.20). CSF P-tau (cutoff ≥ 60 pg/ml): Total– 37.1% (62/167); CDR 0–31.9% (22/69); CDR 0.5–38.3% (31/81); CDR ≥ 1–52.9% (9/17), (Χ^2^ = 2.68, df = 2, p = 0.26); One subject in the CDR 0 group had p-tau below the detection limit. CSF T-tau (cutoff >350 pg/ml): Total– 65.5% (110/168); CDR 0–58.6% (41/70); CDR 0.5–69.1% (56/81); CDR ≥ 1–76.5% (13/17), (Χ^2^ = 2.87, df = 2, p = 0.24).

When CSF biomarker levels were dichotomized into normal vs. abnormal based on aforementioned cutoffs, close to 86% of the entire cohort had abnormal Aβ levels as represented by Aβ42/40 ratios (Fig 1). The proportion of patients with abnormal levels of p-tau and t-tau in the entire cohort were 37% and 65% respectively. The proportion of patients with abnormal CSF p-tau and t-tau increased with higher (worse) CDR scores (Fig 1).

In order to examine the underlying CSF biomarker profiles of hip fracture patients without dementia, biomarkers of patients in the CDR 0 and 0.5 groups were categorized further according to the combination of the A/T/N and corresponding NIA-AA classification system [23]. Among the individuals in the CDR 0 group, 88.6% (62/70) had abnormal CSF biomarker levels. The vast majority had biomarkers suggestive of preclinical AD, and with the remainder in the Suspected Non-Alzheimer’s Pathology (SNAP) category. In the CDR 0.5 group, 98.8% (80/81) had abnormal biomarker levels. Most patients had biomarkers suggestive of prodromal AD, and the remainder were in the MCI-SNAP group (Table 3). All of the individuals (17/17) in the CDR ≥ 1 group had abnormal biomarker levels.

**Table 3:** A/T/N^a^ Classification for CSF biomarkers in non-demented patients with hip fracture.

**CDR 0**	**Normal**^b^	**Preclinical AD**		**SNAP**
n = 70		**Stage 1**	**Stage 2/3**	
	(A-/T-/N-)	(A+/T-/N-)	(A+/T+/N-;	(A-/T+/N-;
			A+/T-/N+	A-/T-/N+;
			A+/T+/N+)	A-/T+/N+)
**Age group (yrs)**		**% (n/subgroup total)**		
65–74, n = 25	16.0(4/25)	24.0(6/25)	44.0(11/25)	16.0(4/25)
75–84, n = 27	11.1(3/27)	29.6(8/27)	59.3(16/27)	0.0(0/27)
85–102, n = 18	5.6(1/18)	38.9(7/18)	44.4(8/18)	11.1(2/18)
**CDR 0.5**	**MCI**	**MCI**		**MCI-SNAP**
n = 81				
	(unlikely due to AD)	(A+/T-/N-; A+/T+/N-; A+/T-/N+;		(A-/T+/N-; A-/T-/N+;
	(A-/T-/N-)	A+/T+/N+)		A-/T+/N+)
**Age group (yrs)**		**% (n/subgroup total)**		
65–74, n = 11	0.0(0/11)	72.7(8/11)		27.3(3/11)
75–84, n = 30	3.3(1/30)	93.3(28/30)		3.3(1/30)
85–102, n = 40	0.0(0/40)	92.5(37/40)		7.5(3/40)

Abbreviations: AD, Alzheimer’s disease; CDR, Clinical Dementia Rating; MCI, Mild Cognitive Impairment; SNAP, Suspected Non-Alzheimer Pathophysiology.

^a^A/T/N system used in this study: A–biomarker of fibrillary Aβ deposition (CSF Aβ42/40 ratio, < 0.08) The Aβ42/Aβ40 ratio was calculated as Aβ42/Aβ40 x 10 as previously has been done [20]; T–biomarker of tau pathology [neurofibrillary tangles] (CSF phosphorylated tau, > 350 pg/ml); N–biomarker of AD-like neurodegeneration or neuronal injury (CSF total tau, ≥ 60 pg/ml).

^b^ A/T/N system mapping to the existing NIA-AA criteria [23].

## Discussion

In this study of older individuals who present with hip fracture requiring surgery, we found a high prevalence of AD pathology evidenced by abnormal levels of CSF biomarkers overall and in those without dementia. Although high prevalence of AD pathology is associated with older age, this finding was also evident in younger hip fracture patients. For example, in our study, 68% of the 65–74 year old cognitively normal (CDR 0) individuals had abnormal Aβ biomarker levels. This is a much higher rate than in the general population, such as rates of 23–32% for abnormal amyloid levels by amyloid-PET or CSF Aβ 42 assays in similarly aged cognitively normal individuals from a recent meta-analysis [24]. Similarly, in a different population based study that categorized cognitively normal individuals by the A/T/N system, the most prevalent group was the normal (A-/T-/N-) biomarker category among individuals between the ages of 50 to late 70’s. In this population, the estimated prevalence of the normal (A-/T-/N-) biomarker category at age 65 was 56% [22]. In contrast, the most prevalent group among cognitively normal 65–74 year olds in our hip fracture study was the preclinical AD category in which abnormal Aβ biomarker levels were accompanied by abnormal levels of either or both p-tau and t-tau. Only 16% in this group had normal (A-/T-/N-) biomarker profile in our study. Taken together, our findings suggest that biomarker evidence of underlying AD or other neurodegenerative pathology is highly prevalent in hip fracture population across all age groups.

The strengths of this study include examination of AD biomarker profile in one of the largest cohorts of well characterized hip fracture patients with CSF collection. In addition, CDR determination in a semi-urgent surgical population provides assessment of global cognitive function in addition to brief preoperative cognitive screening that is usually done in this population. However, important limitations should be acknowledged. One limitation is that some patients on oral anticoagulants were excluded for safety reasons. This excluded many individuals with conditions requiring anticoagulation (e.g. atrial fibrillation), and may have excluded patients whose cognitive impairments were largely due to vascular causes. In addition, due to the semi-urgent nature of the hip fracture repair surgery, it was not possible to use brain imaging (e.g. MRI) to evaluate for vascular disease burden in this study, which is also thought to contribute to gait disorders [25]. We also determined the cutoff for abnormal biomarkers based on population based data as well as previously published cutoffs. We acknowledge that the prevalence of abnormal biomarkers may vary depending on different laboratory assays and different populations, and therefore our findings need to be interpreted with caution. Finally, although we incorporated preoperative assessment of the patient in determining the CDR, it was largely informant based and not the formal process specified in the literature [16]. However, formal preoperative CDR determination is most likely not feasible in a traumatic hip fracture population.

Recently, a large population based study demonstrated that individuals with AD had significantly higher incidence of hip fracture compared to those without AD even after adjusting for age and sex [26]. One of the most important findings from our study is that a large proportion of individuals with hip fracture may have preclinical or prodromal AD. Therefore, higher hip fracture risk may not be limited to only those in the Alzheimer’s dementia stage, but may also extend to those who are in the earlier stages. One of the reasons for the higher hip fracture risk in AD may be the aforementioned association of AD pathology with gait disorders leading to hip fracture. However, another reason for the higher risk of hip fracture in AD may be the role of AD in accelerated bone loss [27]. There is mounting evidence that the brain plays an important role in regulating bone mass, with one of the pathways thought to be the actions of the hypothalamus including the leptinergic-sympathetic axis [28] One study showed that total hypothalamic volume in individuals with AD was associated with bone mineral density (BMD) after adjusting for age and sex [27]. Examination of hip fracture incidence in a larger population based biomarker study may be able to further elucidate the association between AD and hip fracture, and possibly determine if screening for fall risk in preclinical and/or prodromal AD stage may reduce the incidence of hip fracture.

In addition, underlying AD pathology may also have continued repercussions even after the hip fracture is repaired. Perhaps the highly prevalent perioperative cognitive changes that hip fracture patients experience including postoperative delirium and subsequent prolonged rehabilitation, loss of independence, and mortality may be due to brain vulnerability as signaled by underlying AD pathology as well. Future direction will be to examine these outcomes in the context of the CSF biomarker findings. In the future, biomarkers may play a crucial role in preoperatively identifying individuals with preclinical and prodromal AD, and lead to more targeted perioperative interventions to reduce adverse outcomes of hip fracture.

## References

[1] [Anonymous]. 2017 Alzheimer's disease facts and figures. Alzheimer's & Dementia. 2017;13: 325–373. 10.1016/j.jalz.2017.02.001.

[2] Hurd MD, Martorell P, Delavande A, Mullen KJ, Langa KM. Monetary costs of dementia in the United States. N Engl J Med. 2013;368: 1326–1334. 10.1056/NEJMsa1204629 23550670 PMC3959992

[3] Grisso JA, Kelsey JL, Strom BL, Chiu GY, Maislin G, O'Brien LA, et al Risk factors for falls as a cause of hip fracture in women. The Northeast Hip Fracture Study Group. N Engl J Med. 1991;324: 1326–1331. 10.1056/NEJM199105093241905 2017229

[4] Stevens JA, Rudd RA. The impact of decreasing U.S. hip fracture rates on future hip fracture estimates. Osteoporos Int. 2013;24: 2725–2728. 10.1007/s00198-013-2375-9 23632827 PMC4717482

[5] Bentler SE, Liu L, Obrizan M, Cook EA, Wright KB, Geweke JF, et al The aftermath of hip fracture: discharge placement, functional status change, and mortality. Am J Epidemiol. 2009;170: 1290–1299. 10.1093/aje/kwp266 19808632 PMC2781759

[6] Centers for Disease Control and Prevention (CDC). Incidence and costs to Medicare of fractures among Medicare beneficiaries aged > or = 65 years—United States, July 1991-June 1992. MMWR Morb Mortal Wkly Rep. 1996;45: 877–883. 8927007

[7] Buchman AS, Yu L, Wilson RS, Schneider JA, Bennett DA. Association of brain pathology with the progression of frailty in older adults. Neurology. 2013;80: 2055–2061. 10.1212/WNL.0b013e318294b462 23635961 PMC3716398

[8] Del Campo N, Payoux P, Djilali A, Delrieu J, Hoogendijk EO, Rolland Y, et al Relationship of regional brain beta-amyloid to gait speed. Neurology. 2016;86: 36–43. 10.1212/WNL.0000000000002235 26643548 PMC4731288

[9] Tian Q, Resnick SM, Bilgel M, Wong DF, Ferrucci L, Studenski SA. Beta-amyloid burden predicts lower extremity performance decline in cognitively unimpaired older adults. J Gerontol A Biol Sci Med Sci. 2017;72: 716–723. 10.1093/gerona/glw183 27664990 PMC6075426

[10] Wennberg AMV, Savica R, Hagen CE, Roberts RO, Knopman DS, Hollman JH, et al Cerebral amyloid deposition is associated with gait parameters in the Mayo Clinic Study of Aging. J Am Geriatr Soc. 2017;65: 792–799. 10.1111/jgs.14670 27869301 PMC5397339

[11] Stark SL, Roe CM, Grant EA, Hollingsworth H, Benzinger TL, Fagan AM, et al Preclinical Alzheimer disease and risk of falls. Neurology. 2013;81: 437–443. 10.1212/WNL.0b013e31829d8599 23803314 PMC3776538

[12] Schirinzi T, Di Lorenzo F, Sancesario GM, Di Lazzaro G, Ponzo V, Pisani A, et al Amyloid-mediated cholinergic dysfunction in motor impairment related to Alzheimer's Disease. J Alzheimers Dis. 2018;64: 525–532. 10.3233/JAD-171166 29914023

[13] Sieber FE, Neufeld KJ, Gottschalk A, Bigelow GE, Oh ES, Rosenberg PB, et al Effect of Depth of sedation in older patients undergoing hip fracture repair on postoperative delirium: The STRIDE Randomized Clinical Trial. JAMA Surg. 2018. 10.1001/jamasurg.2018.2602 PMC6583071 30090923

[14] Li T, Wieland LS, Oh E, Neufeld KJ, Wang NY, Dickersin K, et al Design considerations of a randomized controlled trial of sedation level during hip fracture repair surgery: a strategy to reduce the incidence of postoperative delirium in elderly patients. Clin Trials. 2017;14: 299–307. 10.1177/1740774516687253 28068834 PMC5446288

[15] Jorm AF. A short form of the Informant Questionnaire on Cognitive Decline in the Elderly (IQCODE): development and cross-validation. Psychol Med. 1994;24: 145–153. 8208879 10.1017/s003329170002691x

[16] Morris JC. The Clinical Dementia Rating (CDR): current version and scoring rules. Neurology. 1993;43: 2412–2414. 10.1212/wnl.43.11.2412-a 8232972

[17] De Meyer G, Shapiro F, Vanderstichele H, Vanmechelen E, Engelborghs S, De Deyn PP, et al Diagnosis-independent Alzheimer disease biomarker signature in cognitively normal elderly people. Arch Neurol. 2010;67: 949–956. 10.1001/archneurol.2010.179 20697045 PMC2963067

[18] Palmqvist S, Zetterberg H, Mattsson N, Johansson P, Alzheimer's Disease Neuroimaging Initiative, Minthon L, et al Detailed comparison of amyloid PET and CSF biomarkers for identifying early Alzheimer disease. Neurology. 2015;85: 1240–1249. 10.1212/WNL.0000000000001991 26354982 PMC4607601

[19] Janelidze S, Zetterberg H, Mattsson N, Palmqvist S, Vanderstichele H, Lindberg O, et al CSF Abeta42/Abeta40 and Abeta42/Abeta38 ratios: better diagnostic markers of Alzheimer disease. Ann Clin Transl Neurol. 2016;3: 154–165. 10.1002/acn3.274 27042676 PMC4774260

[20] Hansson O, Zetterberg H, Buchhave P, Londos E, Blennow K, Minthon L. Association between CSF biomarkers and incipient Alzheimer's disease in patients with mild cognitive impairment: a follow-up study. Lancet Neurol. 2006;5: 228–234. 10.1016/S1474-4422(06)70355-6 16488378

[21] Idland AV, Wyller TB, Stoen R, Eri LM, Frihagen F, Raeder J, et al Preclinical Amyloid-beta and Axonal Degeneration Pathology in Delirium. J Alzheimers Dis. 2017;55: 371–379. 10.3233/JAD-160461 27662296

[22] Jack CR Jr, Wiste HJ, Weigand SD, Therneau TM, Knopman DS, Lowe V, et al Age-specific and sex-specific prevalence of cerebral beta-amyloidosis, tauopathy, and neurodegeneration in cognitively unimpaired individuals aged 50–95 years: a cross-sectional study. Lancet Neurol. 2017;16: 435–444. 10.1016/S1474-4422(17)30077-7 28456479 PMC5516534

[23] Jack CR Jr, Bennett DA, Blennow K, Carrillo MC, Feldman HH, Frisoni GB, et al A/T/N: An unbiased descriptive classification scheme for Alzheimer disease biomarkers. Neurology. 2016;87: 539–547. 10.1212/WNL.0000000000002923 27371494 PMC4970664

[24] Jansen WJ, Ossenkoppele R, Knol DL, Tijms BM, Scheltens P, Verhey FR, et al Prevalence of cerebral amyloid pathology in persons without dementia: a meta-analysis. JAMA. 2015;313: 1924–1938. 10.1001/jama.2015.4668 25988462 PMC4486209

[25] Wennberg AM, Savica R, Mielke MM. Association between Various Brain Pathologies and Gait Disturbance. Dement Geriatr Cogn Disord. 2017;43: 128–143. 10.1159/000456541 28152532 PMC5466166

[26] Tolppanen AM, Taipale H, Tanskanen A, Tiihonen J, Hartikainen S. Comparison of predictors of hip fracture and mortality after hip fracture in community-dwellers with and without Alzheimer's disease—exposure-matched cohort study. BMC Geriatr. 2016;16: 204-016-0383-2. 10.1186/s12877-016-0383-2 PMC5134120 27908278

[27] Loskutova N, Honea RA, Brooks WM, Burns JM. Reduced limbic and hypothalamic volumes correlate with bone density in early Alzheimer's disease. J Alzheimers Dis. 2010;20: 313–322. 10.3233/JAD-2010-1364 20164583 PMC2892930

[28] Zaidi M. Skeletal remodeling in health and disease. Nat Med. 2007;13: 791–801. 10.1038/nm1593 17618270

