# Correction: Abnormal CSF amyloid-β42 and tau levels in hip fracture patients without dementia

**DOI:** 10.1371/journal.pone.0206719

**Published:** 2018-10-26

**Authors:** The PLOS ONE Staff

## Notice of republication

This article was republished on October 16, 2018 to correct for formatting errors in the Materials and methods, Results, and Discussion sections introduced during the typesetting process. The publisher apologizes for these errors. Please download this article again to view the correct version. The originally published, uncorrected article and the republished, corrected article are provided here for reference.

## Supporting information

S1 File Originally published, uncorrected article. (PDF)

S2 File Republished, corrected article. (PDF)

## References

[1] Oh ES, Blennow K, Bigelow GE, Inouye SK, Marcantonio ER, Neufeld KJ, et al (2018) Abnormal CSF amyloid-β42 and tau levels in hip fracture patients without dementia. PLoS ONE 13(9): e0204695 10.1371/journal.pone.0204695 30252906 PMC6155555

